# Supplementary figures and images for: Atmospheric carbon monoxide oxidation is a widespread mechanism supporting microbial survival
Source: ISME J. 2019 Jul 29;13(11):2868–81. doi: 10.1038/s41396-019-0479-8 (PMC6794299; doi:10.1038/s41396-019-0479-8)

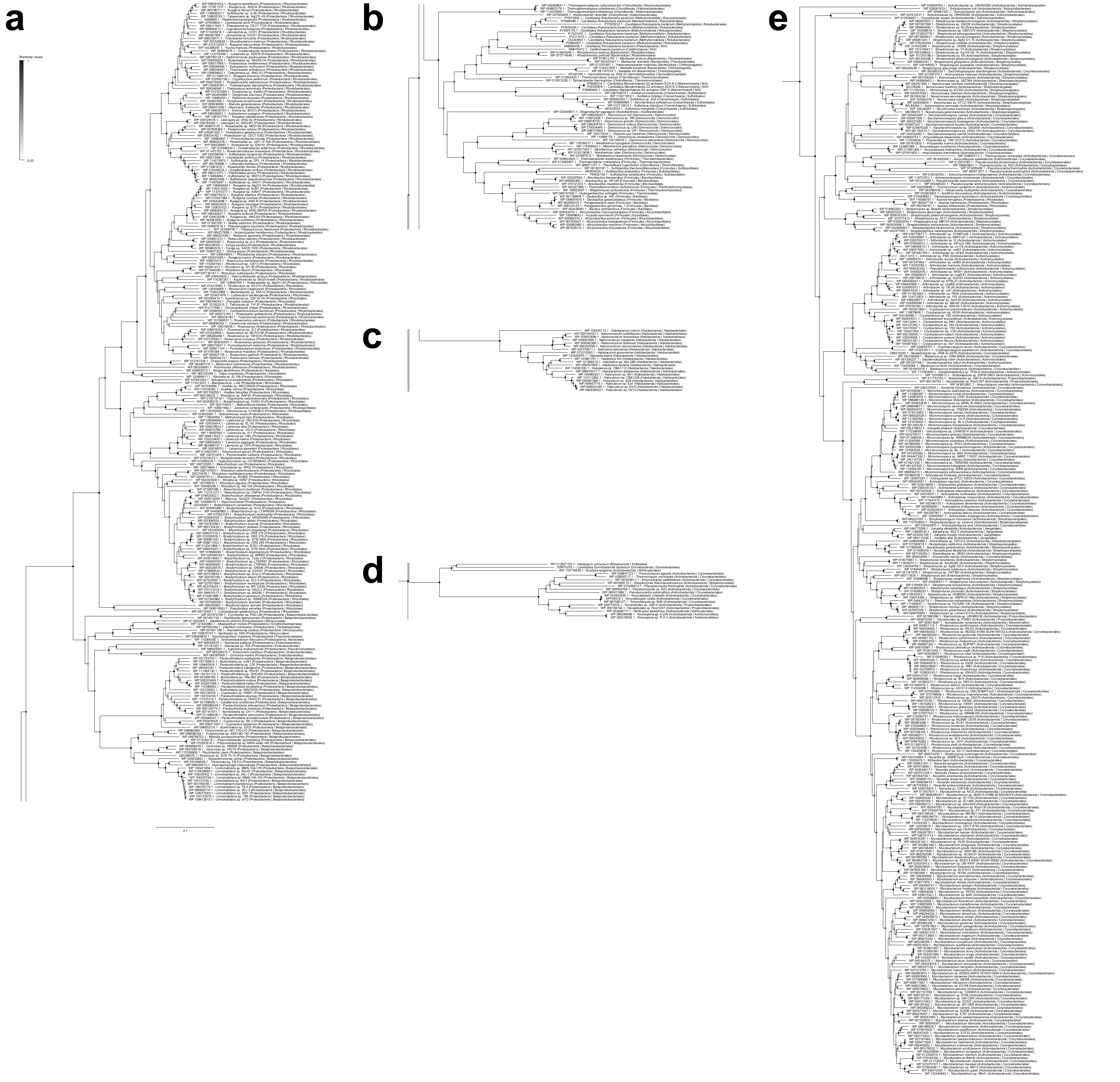

Supplement: Supplementary file 6 — Figure S2 [file 41396_2019_479_MOESM6_ESM.tif]
